# Accuracy of genomic predictions in *Bos indicus* (Nellore) cattle

**DOI:** 10.1186/1297-9686-46-17

**Published:** 2014-02-27

**Authors:** Haroldo HR Neves, Roberto Carvalheiro, Ana M Pérez O’Brien, Yuri T Utsunomiya, Adriana S do Carmo, Flávio S Schenkel, Johann Sölkner, John C McEwan, Curtis P Van Tassell, John B Cole, Marcos VGB da Silva, Sandra A Queiroz, Tad S Sonstegard, José Fernando Garcia

**Affiliations:** 1 UNESP, Universidade Estadual Paulista, Faculdade de Ciências Agrárias e Veterinárias, Jaboticabal, São Paulo 14884-900, Brazil; 2 GenSys Consultores Associados S/C Ltda, Porto Alegre 90680-000, Brasil; 3 Division of Livestock Sciences, Department of Sustainable Agricultural Systems BOKU, University of Natural Resources and Life Sciences, Vienna A-1180, Austria; 4 Centre for Genetic Improvement of Livestock, University of Guelph, Guelph, ON N1G2W1, Canada; 5 Centre for Reproduction and Genomics, AgResearch, Invermay, Mosgiel, New Zealand; 6 United States Department of Agriculture, Agricultural Research Service, Bovine Functional Genomics Laboratory, Beltsville, Maryland 20705, USA; 7 Animal Improvement Programs Laboratory, ARS-USDA, Agricultural Research Service, United States Department of Agriculture, Beltsville, Maryland 20705, USA; 8 Bioinformatics and Animal Genomics Laboratory, Embrapa DairyCattle, Juiz de Fora, Minas Gerais, Brazil; 9 UNESP, Universidade Estadual Paulista, Faculdade de Medicina Veterinária de Araçatuba, Araçatuba, São Paulo 16050-680, Brazil

## Abstract

**Background:**

Nellore cattle play an important role in beef production in tropical systems and there is great interest in determining if genomic selection can contribute to accelerate genetic improvement of production and fertility in this breed. We present the first results of the implementation of genomic prediction in a *Bos indicus* (Nellore) population.

**Methods:**

Influential bulls were genotyped with the Illumina Bovine HD chip in order to assess genomic predictive ability for weight and carcass traits, gestation length, scrotal circumference and two selection indices. 685 samples and 320 238 single nucleotide polymorphisms (SNPs) were used in the analyses. A forward-prediction scheme was adopted to predict the genomic breeding values (DGV). In the training step, the estimated breeding values (EBV) of bulls were deregressed (dEBV) and used as pseudo-phenotypes to estimate marker effects using four methods: genomic BLUP with or without a residual polygenic effect (GBLUP20 and GBLUP0, respectively), a mixture model (Bayes C) and Bayesian LASSO (BLASSO). Empirical accuracies of the resulting genomic predictions were assessed based on the correlation between DGV and dEBV for the testing group.

**Results:**

Accuracies of genomic predictions ranged from 0.17 (navel at weaning) to 0.74 (finishing precocity). Across traits, Bayesian regression models (Bayes C and BLASSO) were more accurate than GBLUP. The average empirical accuracies were 0.39 (GBLUP0), 0.40 (GBLUP20) and 0.44 (Bayes C and BLASSO). Bayes C and BLASSO tended to produce deflated predictions (i.e. slope of the regression of dEBV on DGV greater than 1). Further analyses suggested that higher-than-expected accuracies were observed for traits for which EBV means differed significantly between two breeding subgroups that were identified in a principal component analysis based on genomic relationships.

**Conclusions:**

Bayesian regression models are of interest for future applications of genomic selection in this population, but further improvements are needed to reduce deflation of their predictions. Recurrent updates of the training population would be required to enable accurate prediction of the genetic merit of young animals. The technical feasibility of applying genomic prediction in a *Bos indicus* (Nellore) population was demonstrated. Further research is needed to permit cost-effective selection decisions using genomic information.

## Background

The possibility of accurately predicting the genetic merit of individuals based on their genotypes analyzed by dense single nucleotide polymorphism (SNP) marker panels, a process known as genomic selection (GS) [1,2], is revolutionizing the design and implementation of livestock breeding programs especially for dairy cattle. Schaeffer [3] highlighted the potential benefits of this strategy for dairy cattle in terms of reducing generation intervals, increasing prediction accuracies and selection intensities, reducing breeding organization costs and making it feasible to perform genetic evaluations of difficult-to-measure traits.

The rationale behind genomic selection in livestock is that, given a marker density high enough to cover the entire genome, most of the quantitative trait loci (QTL) will be in high linkage disequilibrium (LD) with some of the markers. Therefore, the sum of all SNP effects (direct genomic value, DGV) will be a good predictor of the genetic merit of selection candidates and will enable selection decisions as soon as the genomic information of those individuals is available [4].

Thanks to the sequencing of the bovine genome [5] and the availability of dense panels of SNP markers, GS has moved from simulation approaches to practical application in the last years. The first successful application of GS was in dairy cattle (Holstein) [4,6] and motivated studies on GS in other breeds and populations [7-9].

Although several previous reports compared statistical methods applied to GS in cattle using the Illumina Bovine 50 K chip (Illumina, San Diego, CA, USA) [7,10,11], only a few studies have carried out similar comparisons using high-density panels, such as the Illumina Bovine HD chip, which contains more than 700 000 SNPs [12]. In addition, most of the studies in this field were carried out using data from *Bos taurus* breeds. While previous studies have investigated the application of GS in purebred and composite populations of *Bos indicus* (Brahman) [13,14], the performance of GS in many other *Bos indicus* populations is unknown.

Nellore cattle are the primary breed used in beef production in tropical systems. Thus, it is expected that genome-enhanced predictions could considerably contribute to improve the efficiency of breeding programs in such systems. Brazil has a large number of well-recorded Nellore animals obtained from several genetic evaluation initiatives [15] that have achieved significant genetic progress for growth traits in the last two decades through conventional selection, although progress for reproduction, meat quality and feed efficiency traits has been less significant during the same period [16].

Our aim was to create the scientific basis for the application of GS to Nellore cattle, by comparing genomic prediction results obtained with four different prediction methods on 15 traits of economic relevance in this breed.

## Methods

### Data

Phenotypic and genotypic data were available for 691 influential Nellore bulls. Genotypes were generated with the Illumina Bovine HD chip (Illumina, San Diego, CA, USA) and only autosomal SNPs with a GenCall (GC) score higher than 0.70 were considered for further analyses. Fifty-four SNP pairs that had the same map coordinates were excluded from the dataset. Quality control of genotypes was carried out through an iterative process using the following SNP selection criteria: call rate (CR) higher than 0.98, minor allele frequency (MAF) higher than 0.02 and p-value for Hardy-Weinberg equilibrium test (HWE) higher than 10^-5^. The SNPs that met these criteria were further screened to interrogate their linkage disequilibrium with syntenic SNPs located within a window of 100 neighboring markers, resulting in only one marker from each pair of highly correlated SNPs (r^2^ > 0.995) remaining in the SNP dataset. Finally, samples showing CR lower than 0.90 were excluded from the analysis. The process was repeated until no further SNPs or samples were excluded, which resulted in a final dataset of 685 bulls with 320 238 SNPs.

Phenotypes were provided by the DeltaGen genetic evaluation program, a commercial beef cattle operation managed as an alliance of breeders distributed across 12 Brazilian states [17]. The estimated breeding values (EBV) from routine genetic evaluations were deregressed and used as dependent variables to estimate SNP effects for 15 traits of economic relevance. These traits included weight and carcass traits, scrotal circumference, gestation length and two selection indexes [See Additional file 1 for detailed trait definitions]. The deregressed proofs (dEBV), as well as their associated reliabilities, were obtained according to the procedure proposed by [18], which removed parent average effects and also accounted for heterogeneous variances [9].

The genotyped individuals included 65 influential older bulls born between 1965 and 1990, while the remaining genotyped animals were younger. The dataset comprised up to four generations of genotyped animals, including 292 son-sire pairs, 139 grandson-grandsire pairs and 51 paternal half-sib families (average size = 4.7). [See Additional file 2 for more information about the age structure of the genotyped animals].

### Genomic prediction design

For each individual trait, a forward prediction scheme was adopted, which splits the dataset into a training (reference) population, that included bulls with EBV accuracies greater than 0.50 in 2007, and a testing population that included bulls that did not have accurate EBV in 2007 but had EBV accuracies greater than 0.50 in 2011.

The sizes of the training and testing datasets differed between traits (Table 1). Most traits were moderately heritable, with heritabilities (h^2^) ranging from 0.25 (score for carcass conformation and finishing precocity at weaning) to 0.49 (gestation length), with an average of about 0.30 (Table 1). Such heritability estimates were based on REML estimates of variance components, obtained using the same database from which the EBV employed in this study were obtained. For all traits, average EBV accuracies were greater than 0.80 and 0.74 in the training and testing sets, respectively.

**Table 1:** **Summary statistics related to the estimated breeding values (EBV) of *Bos indicus *** **(Nellore) bulls included in training and testing sets for 15 traits under forward prediction**^**1**^

**Trait**^**2**^	**h**^**2**^	**Training set**			**Testing set**		
		**N**^**3**^	**Mean EBV (SD)**^**4**^	**Mean accuracy (SD)**^**5**^	**N**^**3**^	**Mean EBV (SD)**^**4**^	**Mean accuracy (SD)**^**5**^
WG	0.26	494	1.60 (5.57)	0.86 (0.12)	187	4.11 (5.17)	0.80 (0.11)
Cw	0.25	472	0.10 (0.31)	0.85 (0.12)	185	0.19 (0.35)	0.79 (0.12)
Pw	0.25	472	-0.03 (0.42)	0.85 (0.12)	184	0.21 (0.42)	0.79 (0.12)
Mw	0.26	473	-0.02 (0.40)	0.85 (0.12)	185	0.20 (0.41)	0.80 (0.11)
Nw	0.27	468	0.02 (0.27)	0.85 (0.12)	188	0.06 (0.23)	0.80 (0.11)
PWG	0.33	473	0.66 (7.58)	0.85 (0.12)	115	2.83 (7.65)	0.81 (0.10)
Cy	0.31	454	0.13 (0.36)	0.84 (0.13)	118	0.29 (0.40)	0.80 (0.11)
Py	0.31	455	-0.06 (0.55)	0.83 (0.13)	117	0.24 (0.53)	0.80 (0.11)
My	0.30	448	-0.05 (0.51)	0.84 (0.12)	121	0.25 (0.50)	0.79 (0.11)
Ny	0.30	443	0.03 (0.30)	0.84 (0.13)	122	0.07 (0.26)	0.79 (0.11)
SCaw	0.40	446	-0.22 (1.21)	0.81 (0.14)	115	-0.15 (1.15)	0.75 (0.12)
BW	0.37	457	0.40 (1.35)	0.86 (0.11)	189	0.15 (1.25)	0.83(0.11)
GL	0.49	307	0.17 (3.25)	0.88 (0.10)	138	-0.77 (4.04)	0.88 (0.10)
WI	-	479	2.74 (13.03)	0.85 (0.12)	185	9.83 (13.10)	0.80 (0.11)
FI	-	465	0.86 (12.13)	0.84 (0.12)	130	8.17 (11.94)	0.77 (0.13)

^1^Training set composed of bulls with accurate EBV in 2007 and testing set composed of remaining bulls with accurate EBV in 2011 but not in 2007; the summary statistics were obtained considering the EBV obtained in either 2007 (training set) or 2011 (testing set); EBV were obtained with BLUP animal models; ^2^WG = weight gain from birth to weaning (about 205 days of age); Cw, Pw, Mw, Nw = visual scores recorded at weaning for carcass conformation, carcass finishing precocity, muscling and navel, respectively; PWG = weight gain from weaning to yearling (at 550 days of age); Cy, Py, My, Ny = visual scores recorded at yearling for carcass conformation, carcass finishing precocity, muscling and navel, respectively; SCaw = scrotal circumference adjusted for age and weight; BW = birth weight; GL = gestation length; WI = weaning index, composed of traits evaluated at weaning; FI = final index, composed of traits evaluated at weaning and yearling (FI) [See Additional file 1 for more details]; ^3^ N = sample size; ^4^Mean EBV(SD) = average (standard deviation) of estimated breeding values (EBV); ^5^Mean accuracy (SD) = average (standard deviation) of EBVs’ accuracies.

In our study, model training was carried out using dEBV based on the 2007 genetic evaluation (dEBV_2007_), while dEBV based on the 2011 genetic evaluation (dEBV_2011_) were used for validation purposes. Using dEBV_2007_ for model training ensured that information of own performance (and/or progeny records) of the testing animals did not contribute to the dEBV of the training set, thus preventing overlapping information between training and testing sets, which could inflate the estimates of predictive ability of GS [19].

Because the dataset included many pairs of closely related animals, the forward prediction scheme resulted in many testing animals having close relatives in the training set. The pattern of relationships between animals in the training and validation sets was consistent across traits [See Additional file 2].

### Statistical methods

The following statistical methods were used in order to estimate SNP effects and direct genomic values (DGV): (i) best linear unbiased prediction (BLUP) using a genomic relationship matrix (GBLUP), (ii) Bayesian regression using a mixture model (Bayes C) and (iii) Bayesian LASSO (BLASSO). All methods only accounted for the allele substitution (additive) effects of the markers, i.e. apart from an overall mean, no other effects (environmental or genetic) were included in the models.

GBLUP model can be described as:

(1) $$y=1_{n}\mu +\mathrm{Zg}+e,$$

where **y** is the vector of dEBV for the respective trait, μ is the location parameter common to all observations, **1**_**n**_ is a vector of 1's, **Z** is the incidence matrix relating genomic breeding values to **y**, **g** is the vector of genomic breeding values and **e** is the vector of random residual terms. It was assumed that **g** ~ N (0,G*σ^2^_g_) and **e** ~ N (0,**R**σ^2^_e_), where G* is a combined relationship matrix and **R** is a diagonal matrix, whose elements account for the differences in the reliabilities of the observations in **y**, similarly as in [20]. The diagonal elements of **R** (R_ii_) were obtained as R_ii_ = (1-*r*_*i*_^*2*^)/(*r*_*i*_^*2*^), where *r*_*i*_^*2*^ is the reliability associated with the i^th^ dEBV, obtained following [18].

The G* matrix is a combined relationship matrix, computed as G* = (1-w)**G** + w**A**, where **G** is the genomic relationship matrix and **A** is the regular numerator relationship matrix, both of order equal to the number of genotyped bulls. **G** was defined as **G** = **MM**'/Σ2p_i_(1-p_i_), in which **M** is the incidence matrix of marker scores whose elements in the i^th^ column are 0-2p_i_, 1-2p_i_ and 2-2p_i_, depending on whether the animal’s genotype was 11, 12 or 22, respectively, and p_i_ is the allele frequency of allele 2 at the i^th^ marker [20].

In the computation of the genomic relationship matrix G*, attributing a weight (w) for pedigree-based relationships is equivalent to fitting residual polygenic effects that are not captured by the markers [21]. After testing different values for w (ranging from 0 to 0.40), Gao et al. [21] reported that w = 0.20 provided the best compromise in terms of reliability and scale of DGV. Since our aim was to investigate the benefit of this strategy, GBLUP predictions were obtained setting w = 0 or w = 0.20, hereafter referred to as GBLUP0 and GBLUP20, respectively.

Theoretically, allele frequencies from the unselected base population should be used to construct **G **[20], which could be estimated after using linear regression to predict gene content (number of copies of a particular allele in a genotype of an individual) of non-genotyped ancestors, based on the available information of genotypes and pedigree [22]. However, there is some evidence that similar accuracies of prediction are obtained using either base population or current allele frequencies [20,23]. Hence, in this study, **G** was constructed using current allele frequencies (computed considering all genotyped animals). The GBLUP method was implemented using the gebv software described in [24]. This formulation of the GBLUP method is equivalent to assuming a normal distribution of SNP effects with constant variance across SNPs [25].

The Bayes C (BayesC) method consisted of fitting a mixture model for SNP effects using the same model equation as in (1), in which **y**, **1**_**n**_, μ, **z**, **g**, and **e** were defined as before, but the elements of vector **g** were calculated for each animal as $\sum_{i=1}^{N}\left(z_{i}a_{i}I_{i}\right),$ where z_i_ is the genotype of the i^th^ marker, coded as the number of copies of the reference allele, *a*_*i*_ is the effect of marker *i*, and *I*_*i*_ is an indicator variable that is equal to 1 if the i^th^ marker has a non-zero effect on the trait and 0 otherwise.

Model parameters were estimated within a Bayesian framework. It was assumed that *a*_*i*_ *~ N(0, σ*^*2*^_*a*_*)* and **e** ~ N (0,**R**σ^2^_e_). Scaled inverse chi-squared distributions, with v degrees of freedom and scale parameter S were assumed for σ^2^_*a*_ and σ^2^_*e*_. Unlike the Bayes B method [2], this mixture model assumes that SNP marker effects are sampled from a single (normal) distribution, instead of estimating marker-specific variances. An arbitrarily small value of 4 was assumed for v, while the scale parameters were derived according to [26]. **R** was defined as described before. A binomial distribution with probability π was assumed for *I*_*i*_ and an informative beta distribution (α = 1.d8, β = 1.d10) was assigned for π (implying that this parameter was kept fixed around 0.01). This method was very similar to that proposed in [26], except that π was assumed to be known, as in [9].

The SNP effects were estimated using the Gibbs sampling algorithm implemented in the GS3 software [27]. A single chain with a length of 100 000 iterations was used. The burn-in period was 20 000 iterations and the thinning interval was 100 iterations.

The model for Bayesian LASSO (BLASSO) was similar to the one in equation (1), except for the assumption about SNP marker effects. This implementation can be understood as a linear mixed model assuming an exponential prior distribution for variances of marker effects.

Originally, the LASSO procedure [28] was a statistical method that combined both variable selection and shrinkage. Legarra et al. [11] proposed an alternative Bayesian implementation of this method, which we used here. Based on the parameterization proposed by these authors, the prior for individual SNP effects (*a*_*i*_) can be represented by:

$$\;P\left(a_{i}|\tau^{2}\right)\sim N\;\left(0,\tau_{i}^{2}\right)\;\text{and}\;P\left(\tau_{i}^{2}|\lambda \right)=\left(\lambda^{2}/2\right)\exp\left(-\lambda^{2}|\tau_{i}^{2}|\right).$$

This parameterization implies that individual variances for each SNP (i.e. τ_i_^2^) are estimated, conditional on a regularization parameter *λ,* which was estimated by using a prior gamma distribution bounded between 0 and 10^7^. Flat priors were assumed for σ^2^_*a*_ and σ^2^_*e*_ and differences in reliabilities of dEBV were accounted for via the matrix **R**, as for the other methods. A single chain with a length of 100 000 iterations was generated using GS3 software. The burn-in period was 20 000 iterations and the thinning interval was 100 iterations.

The programs used to compute genomic predictions handle missing markers internally. In GS3, missing calls for a given marker are set to the population mean for the respective marker, while in the gebv software missing genotypes are inferred using a pedigree-based algorithm. Due to the low frequency of missing genotypes (0.25%) the effects of different imputation procedures are expected to be negligible, as already reported by [29].

### Comparison criteria

The four statistical methods used to derive DGV were evaluated based on comparison of DGV with dEBV_2011_ of animals from the testing set using the following statistics:

(i) Pearson’s correlation between DGV and dEBV_2011_, divided by the average accuracy of dEBV_2011_, was computed as the empirical accuracy of prediction (r_TBV,DGV_). This quantity can be used as a proxy for the correlation of the DGV with the true breeding value [4], which is why it is abbreviated as “r_TBV,DGV_”. The average accuracy of dEBV_2011_ was computed as the average of the dEBV accuracies calculated according to [18].

(ii) the slope of the regression of dEBV_2011_ on DGV for animals in the testing set (b1_dEBV,DGV_) was evaluated to measure the degree of inflation/deflation of genomic predictions, i.e. the scale of the DGV compared to that of dEBV. Estimates of b1_dEBV,DGV_ close to 1 are indicative of predictions that are on scale similar to that of the dEBV.

(iii) the mean squared error of prediction (MSE) between DGV and dEBV of animals in the testing set was used as a measure of the overall fit of each model to the data. Larger estimates of r_TBV,DGV_ are indicative of more reliable predictions and a lower MSE is associated with a better overall fit, including scale.

### Alternative validation designs

In addition to the forward prediction scheme (FORW), two alternative validation strategies were tested for GBLUP20 in order to investigate the impact of the genetic relationship between training and testing sets on the accuracy of genomic predictions in this population. These strategies were based on 5-fold cross-validation that either separated animals in five groups of similar size at random (RAND) or based on minimizing genetic relationships between groups (DIST). For DIST, a k-means algorithm [30] was applied, with the distance matrix built based on the genomic relationships among genotyped animals, similar to [9]. In the case of RAND and DIST, the dEBV generated from the 2011 genetic evaluations were used for both the training and testing steps and the average r_TBV,DGV_ (calculated using the five folds) was used as a proxy for the empirical accuracy of the DGV.

### Impact of relatedness with training set on the accuracy of individual DGV

In order to investigate the extent to which individual accuracies of the DGV of animals in the testing set were influenced by their relatedness with individuals from the training set, under the forward prediction design, different measures of its genomic relatedness with animals in the training set were calculated for each animal in the testing set, based on the genomic relationship matrix (G) used in GBLUP, similar to [31]. The maximum relationship (maxr) and the average of the top 5 (ave5), 10 (ave10), 20 (ave20) and 50 (ave50) relationships between each testing animal and all animals in the training set were calculated. Since GBLUP allowed the calculation of individual DGV accuracies based on elements of the inverse of the coefficient matrix (hereafter, estimated accuracies, or rPEV), the correlations of rPEV with the different measures of relatedness with the training set were determined.

Finally, the empirical accuracies and estimated accuracies (averaged across animals in the testing set) were compared with the analytical expectation for accuracy of genomic predictions, calculated according to a formula proposed by [32] (i.e. Equation 1 in that study). This formula predicts the expected accuracy for an animal without phenotypic information and without close relatives in the training set, as a function of the number of animals in the training set, the heritability of pseudo-phenotypes and effective number of chromosome segments (Me), which was approximated using estimates of genome size (L) and effective population size (Ne), i.e. Me = 2NeL/ln(4NeL). For such calculations, a 30 Morgan genome was assumed, the average reliability of the animals in the training set were considered as the heritability of pseudo-phenotypes, and markers were assumed to capture 80% of the genetic variance for all traits. A value of 120 was adopted for Ne, similar to the estimate obtained by [33] for the population used in this study.

## Results

### Minor allele frequency and linkage disequilibrium

After quality control of the genotyping data (QC), the average (SD) minor allele frequency was 0.226 (0.144) and the average (median) linkage disequilibrium (r^2^) between pairs of adjacent markers was 0.293 (0.164).

### Accuracy of genomic predictions

Empirical accuracies of genomic predictions (r_TBV,DGV_) ranged from 0.17 (navel at weaning) to 0.74 (carcass finishing precocity at yearling). The average empirical accuracy across traits was 0.39 and 0.40 for GBLUP0 and GBLUP20, respectively, and 0.44 for both BayesC and BLASSO (Table 2). For traits measured in both periods, empirical accuracies were from 18% to 61% higher at yearling than at weaning.

**Table 2:** **Empirical accuracies and inflation of genomic predictions obtained for 15 traits of *Bos indicus *** **(Nellore) cattle based on different methods**

	**r(TBV,DGV)**^**1**^				**b1(dEBV,DGV)**^**2**^			
**Trait**^**3**^	**GBLUP0**	**GBLUP20**	**BayesC**	**BLASSO**	**GBLUP0**	**GBLUP20**	**BayesC**	**BLASSO**
WG	0.28	0.27	0.37	0.37	0.79	0.85	1.45	1.39
Cw	0.21	0.18	0.22	0.23	0.85	0.88	1.12	1.10
Pw	0.43	0.45	0.49	0.49	1.08	1.12	1.37	1.35
Mw	0.43	0.44	0.49	0.49	1.09	1.14	1.41	1.39
Nw	0.17	0.17	0.20	0.19	0.75	0.85	1.01	0.99
PWG	0.53	0.56	0.50	0.51	0.92	1.06	1.47	1.43
Cy	0.29	0.30	0.29	0.29	0.98	1.14	1.30	1.26
Py	0.70	0.72	0.74	0.74	1.19	1.24	1.39	1.37
My	0.68	0.69	0.69	0.69	1.13	1.22	1.32	1.30
Ny	0.20	0.20	0.23	0.24	0.94	1.05	1.19	1.19
SCaw	0.68	0.71	0.72	0.72	1.27	1.44	1.68	1.65
BW	0.24	0.24	0.30	0.30	0.57	0.70	0.94	0.91
GL	0.22	0.24	0.36	0.36	0.90	1.09	2.35	2.12
WI	0.30	0.30	0.39	0.39	0.87	0.93	1.39	1.36
FI	0.49	0.51	0.55	0.54	1.01	1.11	1.40	1.37

^1^Accuracies measured as the Pearson’s correlation between direct genomic values (DGV) and deregressed EBV (dEBV) of the bulls in the testing set, r(dEBV,DGV), divided by the average accuracy of dEBV in the testing set; ^2^Inflation of genomic predictions measured by the slope of the regression of dEBV on DGV, b1(dEBV,DGV); The estimates of empirical accuracies and inflation refer to the forward prediction design; ^3^WG = weight gain from birth to weaning (about 205 days of age); Cw, Pw, Mw, Nw = visual scores taken at weaning for carcass conformation, finishing precocity, muscling and navel, respectively; PWG = weight gain from weaning to yearling (about 550 days of age); Cy, Py, My, Ny = visual scores taken at yearling for carcass conformation, finishing precocity, muscling and navel, respectively; SCaw = scrotal circumference adjusted for age and weight; BW = birth weight; GL = gestation length; WI = weaning index, composed by traits evaluated at weaning; FI = final index, composed by traits evaluated at weaning and yearling (FI) [See Additional file 1 for more details].

For most traits, GBLUP20 resulted in slightly greater accuracies than GBLUP0, although this advantage was greater (12%) for gestation length, while for conformation at weaning, GBLUP0 was 13% more accurate than GBLUP20 (Table 2). In general, empirical accuracies of BayesC were very similar to those of BLASSO and superior to those achieved with both implementations of GBLUP. The largest advantage of Bayesian regression methods over GBLUP20 in terms of empirical accuracy was obtained for gestation length (+48%), weight gain from birth to weaning (+35%), conformation at weaning (+25%) and birth weight (+25%). Conversely, GBLUP20 was more accurate than Bayesian regressions for weight gain from weaning to yearling (+9.5%) and for conformation at yearling (+4.5%) (Table 2).

### Scale of genomic predictions and mean squared prediction error (MSE)

The slope of the regression of dEBV on DGV (b1_dEBV,DGV_) was expected to be close to 1, which would indicate that genomic predictions are on a similar scale as the deregressed EBV, i.e. not inflated or deflated. In general, both GBLUP0 and GBLUP20 outperformed the Bayesian regression methods in terms of scale, i.e., for most traits, predictions of DGV obtained with both BayesC and BLASSO were deflated (Table 2). Predictions from GBLUP20 tended to be slightly deflated, while those from GBLUP0 tended to be slightly inflated (Table 2). When averaged across traits, the slope of the regression of dEBV on DGV was equal to 0.96, 1.05, 1.39 and 1.35 for GBLUP0, GBLUP20, BayesC and BLASSO, respectively. However, for birth weight and navel at weaning, BayesC and BLASSO clearly outperformed GBLUP in terms of scale.

For most traits, the overall fit of the model to the data, judged by the mean squared prediction error (MSE), favored both GBLUP methods over the Bayesian regression methods (Table 3). However, for three of the traits (scrotal circumference, birth weight and gestation length), lower estimates of MSE were obtained for Bayes C and Bayesian LASSO (Table 3).

**Table 3:** **Mean squared error (MSE) of genomic predictions for 15 traits**^**2 **^**of *Bos indicus *** **(Nellore) cattle based on different prediction methods**

	**MSE**^**1**^			
**Trait**^**3**^	**GBLUP0**	**GBLUP20**	**BayesC**	**BLASSO**
WG	164.1	165.0	212.6	209.6
Cw	0.7	0.70	0.8	0.9
Pw	0.9	0.9	1.2	1.3
Mw	0.8	0.8	1.1	1.2
Nw	0.3	0.3	0.5	0.7
PWG	194.4	192.6	291.0	335.7
Cy	0.8	0.8	1.2	1.3
Py	0.9	0.9	2.0	2.7
My	0.8	0.8	1.4	1.8
Ny	0.3	0.3	0.6	0.8
SCaw	6.1	6.0	6.0	5.8
BW	5.5	5.4	5.3	5.3
GL	48.9	48.4	47.6	47.5
WI	1029.4	1031.3	1409.1	1602.1
FI	708.5	704.1	1415.0	1568.9

^1^MSE: mean squared prediction error. $\mathrm{MSE}=\frac{1}{N}\sum {\left(\mathrm{DGV}-\mathrm{dEBV}\right)}^{2}$; this statistic was calculated considering the bulls in the testing set, under the forward prediction design; ^2^ WG = weight gain from birth to weaning (about 205 days of age); Cw, Pw, Mw, Nw = visual scores taken at weaning for carcass conformation, finishing precocity, muscling and navel, respectively; PWG = weight gain from weaning to yearling (about 550 days of age); Cy, Py, My, Ny = visual scores taken at yearling for carcass conformation, finishing precocity, muscling and navel, respectively; SCaw = scrotal circumference adjusted for age and weight; BW = birth weight; GL = gestation length; WI: weaning index, composed by traits evaluated at weaning; FI = final index, composed of traits evaluated at weaning and yearling (FI) [See Additional file 1 for more details].

### Individual accuracy of DGV

For most traits, the average accuracy of the DGV (rPEV) was around 0.46, ranging from 0.22 to 0.61 (Table 4). Correlations between accuracies estimated for individuals in the testing set and their relatedness with animals in the training set were strong. The best predictor for this association was the average of the top five relationships between a testing animal and animals in the training set (ave5), for which the average correlation with rPEV across traits was 0.81. The maximum relationship between a testing animal and animals in the training set (maxr) also exhibited a strong association with rPEV (average correlation of 0.78). Across all animals in the testing set, the average maxr and ave5 was equal to 0.35 and 0.20, respectively (Table 4).

**Table 4:** Summary statistics for the accuracy of individual DGV for testing set animals and its association to relatedness with the training set for 15 traits* of *Bos indicus * **(Nellore) cattle**

	**rPEV**^**1**^			**Correlation (rPEV, relatedness)**^**2**^					**Average relatedness**^**3**^	
**Trait**^**3**^	**Average**	**Min**	**Max**	**maxr**	**ave5**	**ave10**	**ave20**	**ave50**	**maxr**	**ave5**
WG	0.47	0.25	0.61	0.81	0.83	0.68	0.52	0.39	0.35	0.19
Cw	0.46	0.25	0.61	0.81	0.83	0.68	0.52	0.39	0.35	0.19
Pw	0.46	0.25	0.61	0.81	0.83	0.68	0.52	0.39	0.35	0.19
Mw	0.46	0.25	0.61	0.81	0.83	0.68	0.52	0.39	0.35	0.19
Nw	0.46	0.24	0.61	0.82	0.82	0.68	0.52	0.39	0.35	0.19
PWG	0.47	0.27	0.61	0.72	0.79	0.66	0.52	0.42	0.36	0.20
Cy	0.47	0.25	0.60	0.71	0.79	0.65	0.52	0.43	0.36	0.20
Py	0.47	0.25	0.60	0.72	0.79	0.65	0.52	0.43	0.36	0.20
My	0.46	0.25	0.60	0.72	0.79	0.65	0.51	0.42	0.35	0.20
Ny	0.46	0.25	0.60	0.72	0.80	0.66	0.52	0.43	0.36	0.20
SCaw	0.45	0.23	0.60	0.71	0.82	0.69	0.55	0.44	0.35	0.20
BW	0.46	0.25	0.61	0.83	0.82	0.67	0.50	0.38	0.35	0.19
GL	0.44	0.22	0.60	0.84	0.84	0.70	0.55	0.48	0.33	0.18
WI	0.46	0.25	0.61	0.81	0.83	0.68	0.52	0.39	0.35	0.19
FI	0.46	0.25	0.60	0.78	0.80	0.65	0.50	0.39	0.35	0.20

^1^Estimated theoretical DGV accuracy (rPEV) calculated based on diagonals of inverse of coefficient matrix in GBLUP20; ^2^association evaluated by the correlation between individual DGV accuracy and each measure of relatedness of testing set animals with training set, calculated either as the maximum relationship (maxr) or as the average of the top 5 (ave5), 10 (ave10), 20 (ave20) or 50 (ave50) relationships between each testing animal and all training set animals; ^3^Averages of relatedness of testing set animals with training set, evaluated through maxr or ave5; *WG = weight gain from birth to weaning (about 205 days of age); Cw, Pw, Mw, Nw = visual scores taken at weaning for carcass conformation, finishing precocity, muscling and navel, respectively; PWG = weight gain from weaning to yearling (about 550 days of age); Cy, Py, My, Ny = visual scores taken at yearling for carcass conformation, finishing precocity, muscling and navel, respectively; SCaw = scrotal circumference adjusted for age and weight; BW = birth weight; GL = gestation length; WI = weaning index, composed by traits evaluated at weaning; FI = final index, composed by traits evaluated at weaning and yearling (FI) [See Additional file 1 for more details].

### Expected accuracies

**Figure 1:**
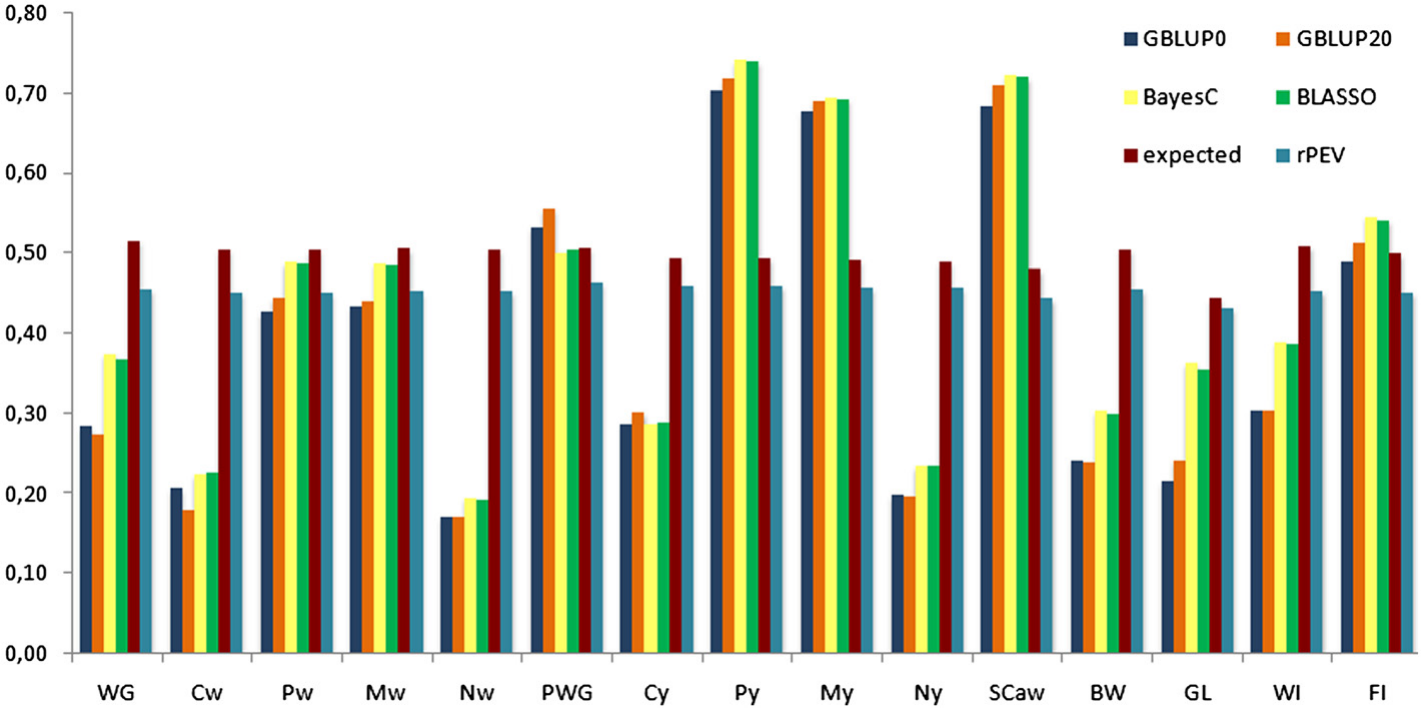
Comparison of empirical and estimated theoretical accuracies (rPEV) with their expectations for 15 traits* in *Bos indicus *(Nellore) cattle. Colored bars indicate: empirical accuracies calculated as the Pearson’s correlation between deregressed proofs in 2011 for the bulls in the testing set and their DGV, divided by the average accuracy of dEBV in the testing set (empirical accuracies were obtained using four methods of prediction: GBLUP20, GBLUP0, Bayes C and Bayesian LASSO); estimated accuracies (rPEV) were calculated by averaging the individual accuracies (obtained based on diagonal elements of the inverse of the coefficient matrix in GBLUP20) across all animals in the testing set; expected accuracies were calculated with the analytical formula proposed by Daetwyler et al. [32]; *WG = weight gain from birth to weaning (about 205 days of age); Cw, Pw, Mw, Nw = visual scores taken at weaning for carcass conformation, finishing precocity, muscling and navel, respectively; PWG = weight gain from weaning to yearling (about 550 days of age); Cy, Py, My, Ny = visual scores taken at yearling for carcass conformation, finishing precocity, muscling and navel, respectively; SCaw = scrotal circumference adjusted for age and weight; BW = birth weight; GL = gestation length; WI = weaning index, composed of traits evaluated at weaning; FI = final index, composed of traits evaluated at weaning and yearling (FI) [See Additional file 1 for more details].

When compared across traits, the mean (SD) of expected accuracies based on Daetwyler’s formula [32] was equal to 0.49 (0.03). In general, although the average empirical accuracies matched their expectations well, values higher than expected were observed for some traits, notably for carcass finishing precocity and muscling evaluated at yearling, as well as for scrotal circumference (Figure 1). In contrast, for conformation at weaning and the navel traits, empirical accuracies were at least 50% lower than their expected values.

### Accuracy of genomic predictions with different validation strategies

**Figure 2:**
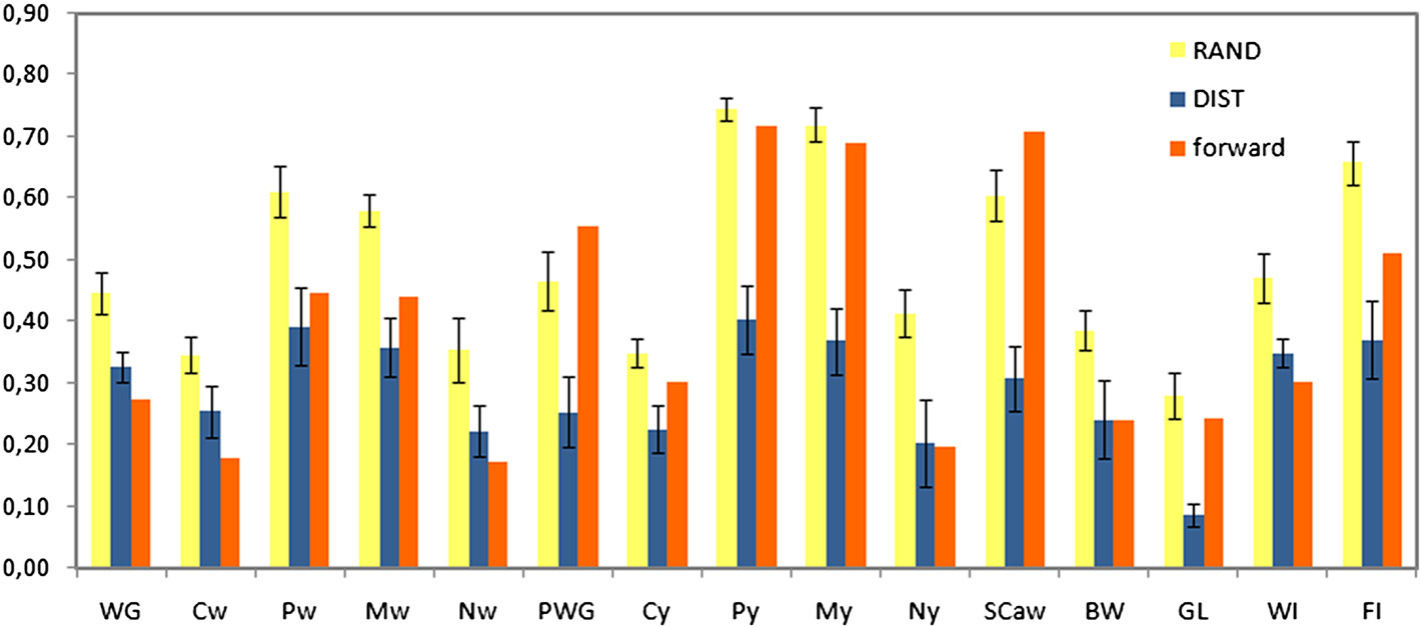
Empirical accuracies of genomic predictions for 15 traits** of *Bos indicus *(Nellore) cattle for different validation strategies*. *forward = training set composed of bulls with highly accurate EBV in 2007 and testing set composed of the remaining bulls (with accurate EBV in 2011); RAND = 5-fold cross-validation (CV), splitting animals randomly into groups of similar size; DIST = 5-fold cross-validation, based on k-means clustering of animals based on their genomic distance (i.e. minimizing inter-groups relationships); empirical accuracies were calculated as the Pearson’s correlation between DGV (obtained with GBLUP20) and deregressed EBV (dEBV) in 2011 for the testing set, divided by the average accuracy of dEBV in the testing set; for the cross-validation strategies (RAND and DIST), the bars and errors bars represent the estimates of means and standard errors obtained in 5-fold CV, respectively; **WG = weight gain from birth to weaning (about 205 days of age); Cw, Pw, Mw, Nw = visual scores taken at weaning for carcass conformation, finishing precocity, muscling and navel, respectively; PWG = weight gain from weaning to yearling (about 550 days of age); Cy, Py, My, Ny = visual scores taken at yearling for carcass conformation, finishing precocity, muscling and navel, respectively; SCaw = scrotal circumference adjusted for age and weight; BW = birth weight; GL = gestation length; WI = weaning index, composed of traits evaluated at weaning; FI = final index, composed by traits evaluated at weaning and yearling (FI) [See Additional file 1 for more details].

Across traits, empirical accuracies were on average 41% smaller for DIST than for the RAND strategy (Figure 2). The extent of relatedness between testing and training animals was evaluated using statistics similar to maxr and ave5 (described previously). For this, both maxr and ave5 were averaged across the testing animals of each fold and a pooled average was calculated based on the averages of the five folds.

The pooled averages of maxr and ave5 were 0.37 and 0.24, respectively, under the RAND strategy and were thus slightly greater than those found for the forward prediction strategy. For DIST, pooled averages of maxr and ave5 were about 2-fold lower (0.20 and 0.12, respectively) than for RAND and the forward prediction strategy.

The empirical accuracies obtained for RAND and DIST cannot be compared directly with those of the forward prediction scheme, since dEBV from the same genetic evaluation (dEBV_2011_) were used for both training and testing animals for RAND and DIST, which is expected to bias the predictive abilities upwards.

Considering all traits, the average proportion of animals in the training set was about 83% for both RAND and DIST, although there was greater variation in fold sizes for DIST. The average size of the training set was slightly smaller for the forward prediction scheme, which could also contribute to slightly smaller empirical accuracies in this strategy.

## Discussion

### Linkage disequilibrium in *Bos indicus* (Nellore) cattle

The pattern of linkage disequilibrium decay in indicine populations differs from that observed in taurine populations [34] and beef cattle have a lower level of LD at the same distance than dairy cattle [35]. The average LD between adjacent markers obtained with the Bovine HD panel in Nellore cattle was similar to the values obtained in Holstein populations with 50 k chips [4,36]. This level of LD is sufficient to achieve accurate genomic predictions in *Bos indicus* (Nellore) cattle [37], provided enough phenotypic information is used to estimate marker effects.

### Genomic prediction methods

Based on the empirical accuracies of prediction, BayesC and BLASSO outperformed the two alternative implementations of GBLUP, with few exceptions. For most traits, GBLUP predictions had smaller MSE and a scale more compatible with that of the deregressed EBV used for validation, when compared to the Bayesian regression methods.

Simulation studies have suggested the superiority of methods based on some sort of variable selection over GBLUP [2,38-40]. This advantage has not been confirmed in many previous studies that compared different methods using real data. In studies using real data, GBLUP performed comparably or better than variable selection methods [4,7,10,11], although there is evidence that substantially higher accuracies can be achieved using variable selection methods for traits that are known to be affected by genes of moderate-to-large effects (e.g. traits affected by *DGAT1*, [6,11]).

The benefit of using variable selection methods is expected to be higher when the number of markers is much greater than the number of genotyped animals. Neither of the previous GS studies on real data contained such large differences between number of animals in the training set and number of genotyped SNPs, thus our study included a scenario for which the use of variable selection methods was expected to provide some benefit. Erbe et al. [12] also confirmed the advantage of a variable selection method (Bayes R) over GBLUP, after analyzing GS in dairy cattle using the same type of high-density panel as we used. These authors suggested that variable selection methods must be used to take full advantage of the increased marker density. The larger empirical accuracies that we obtained with BayesC and BLASSO here corroborate this hypothesis.

Moreover, the considerably greater empirical accuracies that we obtained with BayesC and BLASSO for some of the traits may also suggest the segregation of genes of larger effect for these traits. For instance, a recent GWAS (genome-wide association study) for birth weight, that used data from this same *Bos indicus* population, provided evidence that a region on chromosome 14 had an important effect on this trait [41]; this region had previously been shown to be associated to body size in taurine cattle [42].

The simulation study in [32] provided evidence that the relative advantage of variable selection methods over GBLUP depends on the number of QTL (Nqtl) underlying the trait. When Nqtl is greater than the effective number of chromosome segments, GBLUP should perform equally or better than variable selection methods. In the present study, weight gain from weaning to yearling was the only trait for which a clear advantage in terms of empirical accuracy was observed for GBLUP.

Obtaining individual accuracies and reduced computation time are potential advantages of GBLUP over Bayesian regression methods. The computation time necessary to process all 15 traits took less than one minute with GBLUP, while about two days were required for each of the two Bayesian regression methods (data not shown). The reason for such large differences in computing time is partially due to the fact that the number of genotyped animals is much smaller than the number of markers, and these differences are expected to decline as the number of genotyped animals increases.

### Use of a combined relationship matrix in GBLUP

For most traits, slightly greater empirical accuracy was achieved with GBLUP20 than with GBLUP0, which confirms the results reported by [21]. However, conversely to what these authors indicated, the use of GBLUP20 showed no clear evidence of improvement in the scale of the DGV. This, and the fact that GBLUP0 was slightly more accurate than GBLUP20 for some traits, suggests that the optimal weight (w) for pedigree-based relationships in this alternative implementation of GBLUP may be trait-specific, as pointed out by [43].

### Accuracies of genomic predictions

In the present study, both empirical and estimated accuracies of genomic predictions matched their expectations relatively well but some noteworthy deviances were found. The relatively small number of animals analyzed (n = 685) restricted the formation of training and testing sets to small numbers of individuals, which led to some degree of variation due to sampling, especially when correlations were calculated.

Lower than expected empirical accuracies were estimated for some traits, notably for conformation at weaning and navel. This could be explained by inadequacy of the model used for SNP effect estimation, for instance if the marker density was not sufficiently high to track all genetic variation associated with these traits. In addition, for all traits, expected accuracies were based on the assumption that markers explain 80% of the genetic variance, and the adequacy of this assumption may be trait-specific. Another potential source of noise is related to the fact that the response variables used in model training are prone to prediction errors, the extent of which can also vary across traits.

Further analyses suggested that empirical accuracies greater than expected could be caused by population stratification. As already reported by [41] for this same population, two breeding subgroups were observed in a principal component analysis based on genomic relationships [see Additional file 3]. Other investigations have also shown higher-than-expected accuracies for traits for which EBV means differed significantly between these subgroups (data not shown).

The existence of these subgroups is consistent with two different artificial selection criteria that were applied in this population in the past 20 years [41]. The major differences between such selection criteria consist of largely different emphases on the traits for which we observed higher-than-expected accuracies in the present study (i.e. carcass finishing precocity at yearling, muscling at yearling and scrotal circumference).

The results of this study seemed to confirm the association reported by [31] between the accuracy of individual DGV and the relatedness of testing and training animals, although the strength of this association was lower in the present study. The authors of [31] found that the average of the top 10 relationships with training animals (ave10) was a better predictor of estimated accuracies than the maximum relationship, while the opposite was observed in this study.

For some traits, the average of the estimated individual accuracies was consistent with the empirical accuracies, while this did not hold for other traits. Clark et al. [31] also showed that, while estimated and empirical accuracies agreed well for simulated data and for eye muscle depth in Merino sheep, larger differences between these two sets of accuracies were found for live weight in the same population.

Reasonable evidence for an association between the relationship of the animals in the testing set and the training set and the accuracies of DGV was found, which confirmed the report by [38]. The cross-validation strategies applied in the present study (RAND and DIST) indicated that the same association also holds for empirical accuracies. In this context, when comparing RAND and DIST, we observed that empirical accuracies nearly halved with a 2-fold decrease in average relatedness between testing and training set animals. A consequence of this observation is the possibility of evaluating to which extent the relationship between selection candidates and training animals would affect the accuracy of genomic predictions. Based on the estimates of relationships calculated according to top5 and maxr under the forward prediction scheme, it is expected that the accuracy of DGV prediction will not differ much from values reported here, given that the sire or a few half-sibs are included in the training set for most selection candidates in this population. Thus, application of GS in this population requires a dynamic training set, because recurrent inclusion of new sires in the training population is necessary to enhance predictions of the genetic merit of young animals [9].

### Scale of genomic predictions

Although our study mainly focused on the accuracies of genomic predictions, depending on the selection scheme, the scale of predictions should be a matter of concern, especially to determine whether DGV can be compared to traditional EBV from routine evaluations. For example, in situations in which both progeny-tested and newborn animals are selection candidates, an artificial overestimation of the genetic trend would lead to undue exaggeration of DGV over traditional EBV, as discussed by [44].

Although Bayes C and BLASSO were more accurate than GBLUP for most traits in the present study, these Bayesian regression methods tended to generate deflated predictions. Previous studies have found large differences in the scale of genomic predictions obtained using Bayesian regression. Some of these studies do not agree with the trend of deflation we observed here (e.g. [12,45]), while other methods similar to BayesC and BLASSO also resulted in deflated predictions for some traits analyzed [9,11,46]. This variation in scale may be related to differences inherent to the data analyzed (e.g. the extent to which training animals were pre-selected) and to differences in the implementation of the methods. Future studies should investigate whether including a residual polygenic effect in these Bayesian regression models could improve the scale of genomic predictions, as suggested by [45].

### Future work

Because the selection candidates in this population have own performance data recorded before selection decisions take place, the accuracy of traditional EBV based on own performance could be a suitable reference to evaluate the gain in accuracy that can be attributed to GS. Unfortunately, this information was only available for a small subset of the testing animals in our study, due to the fact that a considerable proportion of the animals were born and had own performance data recorded within other breeding programs, although they had enough progeny recorded in the dataset available for this study to obtain accurate EBV in 2011. A proper comparison between empirical accuracies of traditional EBV and DGV should be carried out as soon as more information is available. In addition, DGV accuracies are expected to increase when more animals are genotyped.

Another topic that deserves further investigation is the identification of an optimal marker density for genomic prediction in the population analyzed. Theoretically, a higher marker density is expected to increase the accuracy of genomic predictions, due to stronger LD between markers and QTL [47]. Previous studies that compared genomic predictions obtained with high-density (~777 000 markers, HD) and medium-density panels (~54 000 markers, 54 k) in *Bos taurus* breeds reported only a marginal increase in accuracies when using high-density panels [12,47]. Because the size of the reference population in this study is small, the possible benefits of an increased marker density could be counterbalanced by an increase in the number of unknown parameters to be estimated, as previously suggested by [47]. For instance, in a Jersey population, when genomic predictions were obtained with a training set of size comparable to that of the present study, the accuracy of the DGV decreased slightly when moving from 54 k to HD [12]. The relative benefit of genomic predictions obtained at different marker densities will be evaluated when more information is available.

While these initial results seem to confirm the technical feasibility of applying genomic selection in a *Bos indicus* (Nellore) beef cattle population, further work is needed on the design of breeding schemes for this particular breed. In this context, imputation methods will probably play an important role to improve cost-effectiveness of this technology, as suggested by [48].

## Conclusions

The technical feasibility of applying genomic prediction in a *Bos indicus* (Nellore) population was demonstrated, although further research on its implementation in breeding schemes is necessary to enable more cost-effective selection decisions using genomic information. Bayesian regression models (Bayes C and BLASSO) were more accurate than GBLUP for most traits and are of interest for future applications of genomic selection in this population, but further improvements are needed to reduce deflation of the predictions obtained with such methods. The accuracies of genomic predictions depended on the extent of relatedness between training and testing set animals, which means that recurrent updates of the training population are necessary to enhance predictions of the genetic merit of young animals.

## Competing interests

The authors declare that they have no competing interests.

## Authors’ contributions

JFG and TSS conceived and JFG led the coordination of the study. RC, FSS, JS, JCM, JBC, CPVT, MVGBS and TSS contributed to the study design and coordination. ASC directed the genotyping work. RC, HHRN, AMPO and YTU led the data analysis. RC, HHRN and JFG led the manuscript preparation. FSS, YTU, TSS, CPVT, JBC, AMPO, JCM, JS, ASC, SAQ and MVGBS contributed to draft the manuscript. All authors read and approved the final manuscript.

## Supplementary Material

Additional file 1 **Trait definition.** Description of the traits considered in the analyses. Click here for file

Additional file 2 **Age structure and relationship of the genotyped bulls.** Description: Details about age structure and relationships between the genotyped animals. Click here for file

Additional file 3 **Principal component analysis of the genomic relationships among the genotyped bulls.** Description: Plot of the first two principal components of the genomic relationships among the genotyped bulls, evidencing two subgroups of the sampled *Bos indicus* (Nellore) population. Click here for file

## References

[B1] Nejati-Javaremi A Smith C Gibson JP Effect of total allelic relationship on accuracy of evaluation and response to selection J Anim Sci 1997 75 1738 1745 9222829 10.2527/1997.7571738x

[B2] Meuwissen THE Hayes BJ Goddard ME Prediction of total genetic value using genome-wide dense marker maps Genetics 2001 157 1819 1829 11290733 10.1093/genetics/157.4.1819 PMC1461589

[B3] Schaeffer LR Strategy for applying genome-wide selection in dairy cattle J Anim Breed Genet 2006 123 218 223 10.1111/j.1439-0388.2006.00595.x 16882088

[B4] Hayes BJ Bowman PJ Chamberlain AJ Goddard ME Invited review: genomic selection in dairy cattle: progress and challenges J Dairy Sci 2009 92 433 443 10.3168/jds.2008-1646 19164653

[B5] Elsik CG Tellam RL Worley KC Gibbs RA Muzny DM Weinstock GM Adelson DL Eichler EE Elnitski L Guigó R Hamernik DL Kappes SM Lewin HA Lynn DJ Nicholas FW Reymond A Rijnkels M Skow LC Zdobnov EM Schook L Womack J Alioto T Antonarakis SE Astashyn A Chapple CE Chen HC Chrast J Câmara F Ermolaeva O Henrichsen CN Bovine Genome Sequencing and Analysis Consortium The genome sequence of taurine cattle: a window to ruminant biology and evolution Science 2009 324 522 528 19390049 10.1126/science.1169588 PMC2943200

[B6] VanRaden PM Van Tassell CP Wiggans GR Sonstegard TS Schnabel RD Taylor JF Schenkel FS Invited review: reliability of genomic predictions for North American Holstein bulls J Dairy Sci 2009 92 16 24 10.3168/jds.2008-1514 19109259

[B7] Luan T Woolliams JA Lien S Kent M Svendsen M Meuwissen THE The accuracy of genomic selection in Norwegian Red cattle assessed by cross-validation Genetics 2009 183 1119 1126 10.1534/genetics.109.107391 19704013 PMC2778964

[B8] Weigel KA de Los CG Vazquez AI Rosa GJM Gianola D Van Tassell CP Accuracy of direct genomic values derived from imputed single nucleotide polymorphism genotypes in Jersey cattle J Dairy Sci 2010 93 5423 5435 10.3168/jds.2010-3149 20965358

[B9] Saatchi M McClure MC McKay SD Rolf MM Kim J Decker JE Taxis TM Chapple RH Ramey HR Northcutt SL Bauck S Woodward B Dekkers JCM Fernando RL Schnabel RD Garrick DJ Taylor JF Accuracies of genomic breeding values in American Angus beef cattle using K-means clustering for cross-validation Genet Sel Evol 2011 43 40 10.1186/1297-9686-43-40 22122853 PMC3250932

[B10] Moser G Tier B Crump RE Khatkar MS Raadsma HW A comparison of five methods to predict genomic breeding values of dairy bulls from genome-wide SNP markers Genet Sel Evol 2009 41 56 10.1186/1297-9686-41-56 20043835 PMC2814805

[B11] Legarra A Robert-Granié C Croiseau P Guillaume F Fritz S Improved Lasso for genomic selection Genet Res (Camb) 2011 93 77 87 10.1017/S0016672310000534 21144129

[B12] Erbe M Hayes BJ Matukumalli LK Goswami S Bowman PJ Reich CM Mason BA Goddard ME Improving accuracy of genomic predictions within and between dairy cattle breeds with imputed high-density single nucleotide polymorphism panels J Dairy Sci 2012 95 4114 4129 10.3168/jds.2011-5019 22720968

[B13] Elzo MA Lamb GC Johnson DD Thomas MG Misztal I Rae DO Martinez CA Wasdin JG Driver JD Genomic-polygenic evaluation of Angus-Brahman multibreed cattle for feed efficiency and postweaning growth using the Illumina 3K chip J Anim Sci 2012 90 2488 2497 10.2527/jas.2011-4730 22785165

[B14] Bolormaa S Pryce JE Kemper K Savin K Hayes BJ Barendse W Zhang Y Reich CM Mason BA Bunch RJ Harrison BE Reverter A Herd RM Tier B Graser H-U Goddard ME Accuracy of prediction of genomic breeding values for residual feed intake and carcass and meat quality traits in Bos taurus, *Bos indicus*, and composite beef cattle J Anim Sci 2013 91 3088 3104 10.2527/jas.2012-5827 23658330

[B15] Montaldo HH Casas E Sterman Ferraz JB Vega-Murillo VE Roman-Ponce SI Opportunities and challenges from the use of genomic selection for beef cattle breeding in Latin America Anim Front 2012 2 23 29 10.2527/af.2011-0029

[B16] Garcia JF Carmo AS Utsunomiya YT Neves HHR Carvalheiro R Van Tassell CP Sonstegard TS Silva MVGB Souto MC, Kann MG How Bioinformatics Enables Livestock Applied Sciences in the Genomic Era Lecture Notes in Computer Science (Advances in Bioinformatics and Computational Biology) 2012 Berlin: Springer 192 201

[B17] Conexão Delta G Sumário de avaliação de reprodutores—Gensys Consultores Associados S/C Ltda [http://www.gensys.com.br/home/win_sumarios.php?id_sumario=56]

[B18] Garrick DJ Taylor JF Fernando RL Deregressing estimated breeding values and weighting information for genomic regression analyses Genet Sel Evol 2009 41 55 10.1186/1297-9686-41-55 20043827 PMC2817680

[B19] Amer PR Banos G Implications of avoiding overlap between training and testing data sets when evaluating genomic predictions of genetic merit J Dairy Sci 2010 93 3320 3330 10.3168/jds.2009-2845 20630248

[B20] VanRaden PM Efficient methods to compute genomic predictions J Dairy Sci 2008 91 4414 4423 10.3168/jds.2007-0980 18946147

[B21] Gao H Christensen OF Madsen P Nielsen US Zhang Y Lund MS Su G Comparison on genomic predictions using three GBLUP methods and two single-step blending methods in the Nordic Holstein population Genet Sel Evol 2012 44 8 10.1186/1297-9686-44-8 22455934 PMC3400441

[B22] Gengler N Mayeres P Szydlowski M A simple method to approximate gene content in large pedigree populations: application to the myostatin gene in dual-purpose Belgian Blue cattle Animal 2007 1 21 28 10.1017/S1751731107392628 22444206

[B23] Aguilar I Misztal I Johnson DL Legarra A Tsuruta S Lawlor TJ Hot topic: a unified approach to utilize phenotypic, full pedigree, and genomic information for genetic evaluation of Holstein final score J Dairy Sci 2010 93 743 752 10.3168/jds.2009-2730 20105546

[B24] Sargolzaei M Schenkel FS VanRaden PM gebv: Genomic breeding value estimator for livestock Technical report to the Dairy Cattle Breeding and Genetics Committee 2009 Guelph: University of Guelph

[B25] Goddard ME Genomic selection: prediction of accuracy and maximisation of long term response Genetica 2009 136 245 257 10.1007/s10709-008-9308-0 18704696

[B26] Habier D Fernando RL Kizilkaya K Garrick DJ Extension of the Bayesian alphabet for genomic selection BMC Bioinformatics 2011 12 186 10.1186/1471-2105-12-186 21605355 PMC3144464

[B27] Legarra A Ricard A Filangi O GS3 software: Genomic Selection, Gibbs Sampling and Gauss-Seidel[http://genoweb.toulouse.inra.fr/~alegarra/manualgs3_last.pdf]

[B28] Tibshirani R Regression shrinkage and selection via the lasso J R Stat Soc B 1996 58 267 288

[B29] Zapata-Valenzuela J Whetten RW Neale D McKeand S Isik F Genomic estimated breeding values using genomic relationship matrices in a cloned population of loblolly pine G3 2013 3 909 916 2013 23585458 10.1534/g3.113.005975 PMC3656736

[B30] Hartigan JA Wong MA Algorithm AS 136: a k-means clustering algorithm Appl Stat 1979 28 100 108 10.2307/2346830

[B31] Clark SA Hickey JM Daetwyler HD van der Werf JHJ The importance of information on relatives for the prediction of genomic breeding values and the implications for the makeup of reference data sets in livestock breeding schemes Genet Sel Evol 2012 44 4 10.1186/1297-9686-44-4 22321529 PMC3299588

[B32] Daetwyler HD Pong-Wong R Villanueva B Woolliams JA The impact of genetic architecture on genome-wide evaluation methods Genetics 2010 185 1021 1031 10.1534/genetics.110.116855 20407128 PMC2907189

[B33] Brito FV Sargolzaei M Braccini Neto J Cobuci JA Schenkel FS Pedigree analysis in a large Brazilian Nellore herd Proceedings of the 9th World Congress on Genetics Applied to Livestock Production: 1-6 August 2010 2010 Leipzig: German Society for Animal Science PP1 PP57

[B34] Pérez O’Brien AM Garcia JF Carvalheiro R Neves HHR Van Tassell CP Sonstegard TS Utsunomiya YT Mc Ewan JC Solkner J Comparing linkage disequilibrium between taurine and indicine cattle with a high density SNP chip Book of Abstracts of the 63rd Annual Meeting of the European Association for Animal Production: 27-31 August 2012 2012 Bratislava: Wageningen Academic Publishers 9

[B35] McKay SD Schnabel RD Murdoch BM Matukumalli LK Aerts J Coppieters W Crews D Dias Neto E Gill CA Gao C Mannen H Stothard P Wang Z Van Tassell CP Williams JL Taylor JF Moore SS Whole genome linkage disequilibrium maps in cattle BMC Genet 2007 8 74 17961247 10.1186/1471-2156-8-74 PMC2174945

[B36] Sargolzaei M Schenkel FS Jansen GB Schaeffer LR Extent of linkage disequilibrium in Holstein cattle in North America J Dairy Sci 2008 91 2106 2117 10.3168/jds.2007-0553 18420642

[B37] Espigolan R Baldi F Boligon AA Souza FR Gordo DG Tonussi RL Cardoso DF Oliveira HN Tonhati H Sargolzaei M Schenkel FS Carvalheiro R Ferro JA Albuquerque LG Study of whole genome linkage disequilibrium in Nellore cattle BMC Genomics 2013 14 305 10.1186/1471-2164-14-305 23642139 PMC3662636

[B38] Habier D Fernando RL Dekkers JCM The impact of genetic relationship information on genome-assisted breeding values Genetics 2007 177 2389 2397 18073436 10.1534/genetics.107.081190 PMC2219482

[B39] Solberg TR Sonesson AK Woolliams JA Meuwissen THE Genomic selection using different marker types and densities J Anim Sci 2008 86 2447 2454 10.2527/jas.2007-0010 18407980

[B40] Clark SA Hickey JM Van Der Werf JHJ Different models of genetic variation and their effect on genomic evaluation Genet Sel Evol 2011 43 18 10.1186/1297-9686-43-18 21575265 PMC3114710

[B41] Utsunomiya YT Do Carmo AS Carvalheiro R Neves HHR Matos MC Zavarez LB Perez O’Brien AM Sölkner J McEwan JC Cole JB Van Tassel CP Schenkel FS Silva MVGB Porto Neto LR Sonstegard TS Garcia JF Genome-wide association study for birth weight in Nellore cattle points to previously described orthologous genes affecting human and bovine height BMC Genet 2013 14 52 23758625 10.1186/1471-2156-14-52 PMC3683327

[B42] Karim L Takeda H Lin L Druet T Arias JA Baurain D Cambisano N Davis SR Farnir F Grisart B Harris BL Keehan MD Littlejohn MD Spelman RJ Georges M Coppieters W Variants modulating the expression of a chromosome domain encompassing PLAG1 influence bovine stature Nat Genet 2011 43 405 413 10.1038/ng.814 21516082

[B43] Liu Z Seefried FR Reinhardt F Rensing S Thaller G Reents R Impacts of both reference population size and inclusion of a residual polygenic effect on the accuracy of genomic prediction Genet Sel Evol 2011 43 19 10.1186/1297-9686-43-19 21586131 PMC3107172

[B44] Vitezica ZG Aguilar I Misztal I Legarra A Bias in genomic predictions for populations under selection Genet Res (Camb) 2011 93 357 366 10.1017/S001667231100022X 21767459

[B45] Duchemin SI Colombani C Legarra A Baloche G Larroque H Astruc JM Barillet F Robert-Granié C Manfredi E Genomic selection in the French Lacaune dairy sheep breed J Dairy Sci 2012 95 2723 2733 10.3168/jds.2011-4980 22541502

[B46] Colombani C Legarra A Fritz S Guillaume F Croiseau P Ducrocq V Robert-Granié C Application of Bayesian least absolute shrinkage and selection operator (LASSO) and BayesCπ methods for genomic selection in French Holstein and Montbéliarde breeds J Dairy Sci 2013 96 575 591 10.3168/jds.2011-5225 23127905

[B47] Su G Brondum RF Ma P Guldbrandtsen B Aamand GP Lund MS Comparison of genomic predictions using medium-density (54000) and high-density (777000) single nucleotide polymorphism marker panels in Nordic Holstein and Red Dairy Cattle populations J Dairy Sci 2012 95 4657 4665 10.3168/jds.2012-5379 22818480

[B48] VanRaden PM O’Connell JR Wiggans GR Weigel KA Genomic evaluations with many more genotypes Genet Sel Evol 2011 43 10 10.1186/1297-9686-43-10 21366914 PMC3056758

