# Supplementary material for: Accuracy of genomic predictions in Bos indicus (Nellore) cattle
Source: Genet Sel Evol. 2014 Feb 27;46(1):17. doi: 10.1186/1297-9686-46-17 (PMC4014866; doi:10.1186/1297-9686-46-17)
Supplement: Additional file 1 — Trait definition. Description of the traits considered in the analyses. [file 1297-9686-46-17-S1.pdf]

Additional file 1

Description of the traits considered in the analyses

All traits were based on measurements recorded either at birth, weaning (at 205 days of age) or yearling (at 550 days of age). Weaning traits included weight gain from birth to weaning (WG) and visual scores for carcass conformation (Cw), carcass finishing precocity (Pw), muscling (Mw) and navel (Nw). Yearling traits included weight gain from weaning to yearling (PWG) and scores for conformation (Cy), carcass finishing precocity (Py), muscling (My) and navel (Ny).

Scrotal circumference (in cm) was also measured at yearling and analyzed after adjustment for age and weight (SCaw).

Carcass conformation, carcass finishing precocity and muscling were based on recorded visual scores assigned in a discrete scale, ranging from 1 to 5, relative to the animals of the same management group (the higher the score, more accentuated the presence of the trait).

Scores for navel were assigned based on an absolute scale ranging from 1 to 5. Higher scores for navel are undesirable because they are associated with higher probability of injury in this region.

Birth weight (BW) and gestation length (GL) were mostly used as predictors of calving ease.

Two selection indexes were considered: 1) weaning index (WI) – calculated as a weighted sum of standardized EPDs for weight gain from birth to weaning, expressed as days to gain 160 kg (D160) (Ortiz Peña et al., 2004), Cw, Pw and Mw, with weightings equal to 0.60 (D160), 0.08 (Cw), 0.16 (Pw) and 0.16 (Mw), respectively and 2) final index (FI) – calculated as a weighted sum of standardized EPDs for the same traits considered in the WI plus the traits days to gain 240 kg from weaning to yearling (D240), Cy, Py, My and SCaw, with weightings equal to 0.23 (D160), 0.04 (Cw), 0.08 (Pw), 0.08 (Mw), 0.23 (D240), 0.04 (Cy), 0.08 (Py), 0.08 (My) and 0.14 (SCaw).
