# Supplementary material for: Accuracy of genomic predictions in Bos indicus (Nellore) cattle
Source: Genet Sel Evol. 2014 Feb 27;46(1):17. doi: 10.1186/1297-9686-46-17 (PMC4014866; doi:10.1186/1297-9686-46-17)
Supplement: Additional file 2 — Age structure and relationship of the genotyped bulls. Description: Details about age structure and relationships between the genotyped animals. [file 1297-9686-46-17-S2.pdf]

Additional file 2

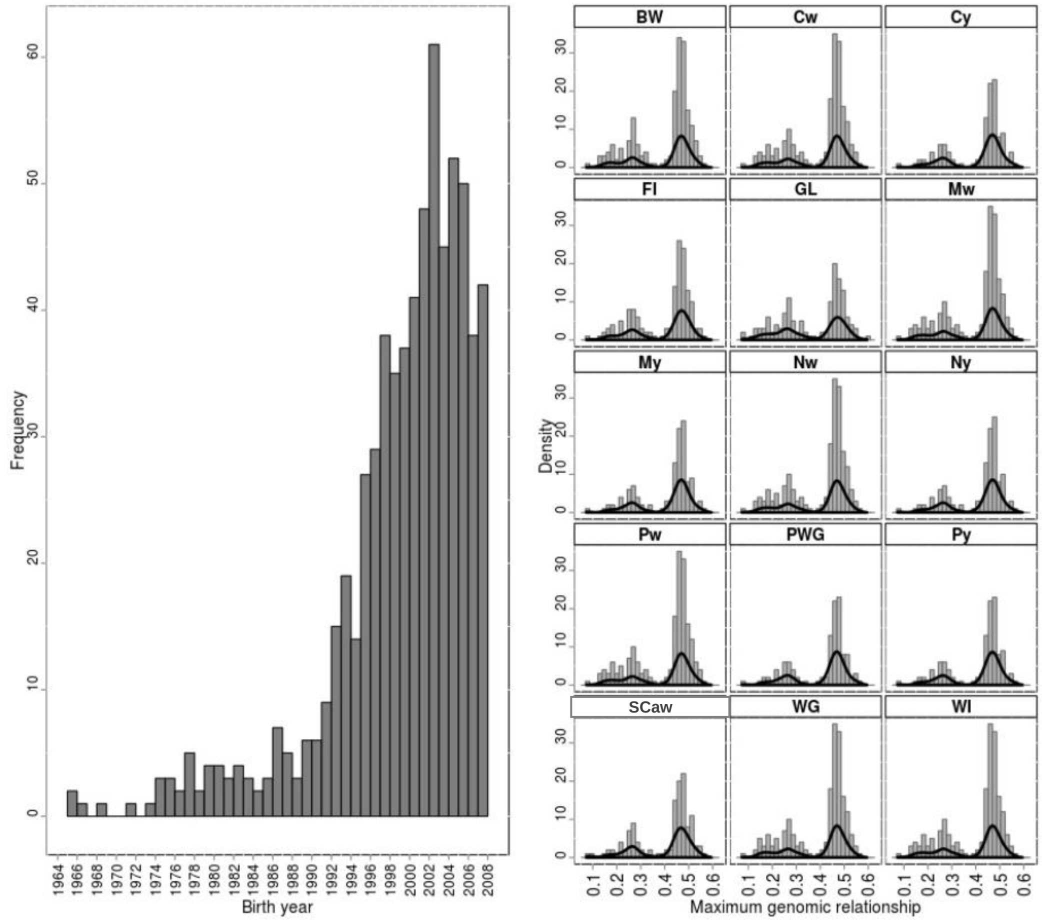

Age structure and relationship of the genotyped bulls
Left) Frequency of birth year of the genotyped bulls.
Right) Maximum genomic relationship[*] (maxr) between animals of testing set and training set, according to the trait analyzed[**].

[*]For each trait analyzed under the forward prediction scheme, the maximum genomic relationship between each testing animal and animals in the training set was computed. The histogram (grey) and the density (black line) of maxr are plotted according to each trait analyzed under the forward prediction scheme.

[**]WG: weight gain from birth to weaning (about 205 days of age); Cw, Pw, Mw, Nw: visual scores taken at weaning for carcass conformation, finishing precocity, muscling and navel, respectively; PWG: weight gain from weaning to yearling (about 550 days of age); Cy, Py, My, Ny: visual scores taken at yearling for carcass conformation, finishing precocity, muscling and navel, respectively; SCaw: scrotal circumference adjusted for age and weight. BW: birth weight; GL: gestation length; WI: weaning index, composed by traits evaluated at weaning; FI: final index, composed by traits evaluated at weaning and yearling (FI) (See Additional file 1 for more details).
