# Supplementary material for: Accuracy of genomic predictions in Bos indicus (Nellore) cattle
Source: Genet Sel Evol. 2014 Feb 27;46(1):17. doi: 10.1186/1297-9686-46-17 (PMC4014866; doi:10.1186/1297-9686-46-17)
Supplement: Additional file 3 — Principal component analysis of the genomic relationships among the genotyped bulls. Description: Plot of the first two principal components of the genomic relationships among the genotyped bulls, evidencing two subgroups of the sampled Bos indicus (Nellore) population. [file 1297-9686-46-17-S3.pdf]

Additional file 3

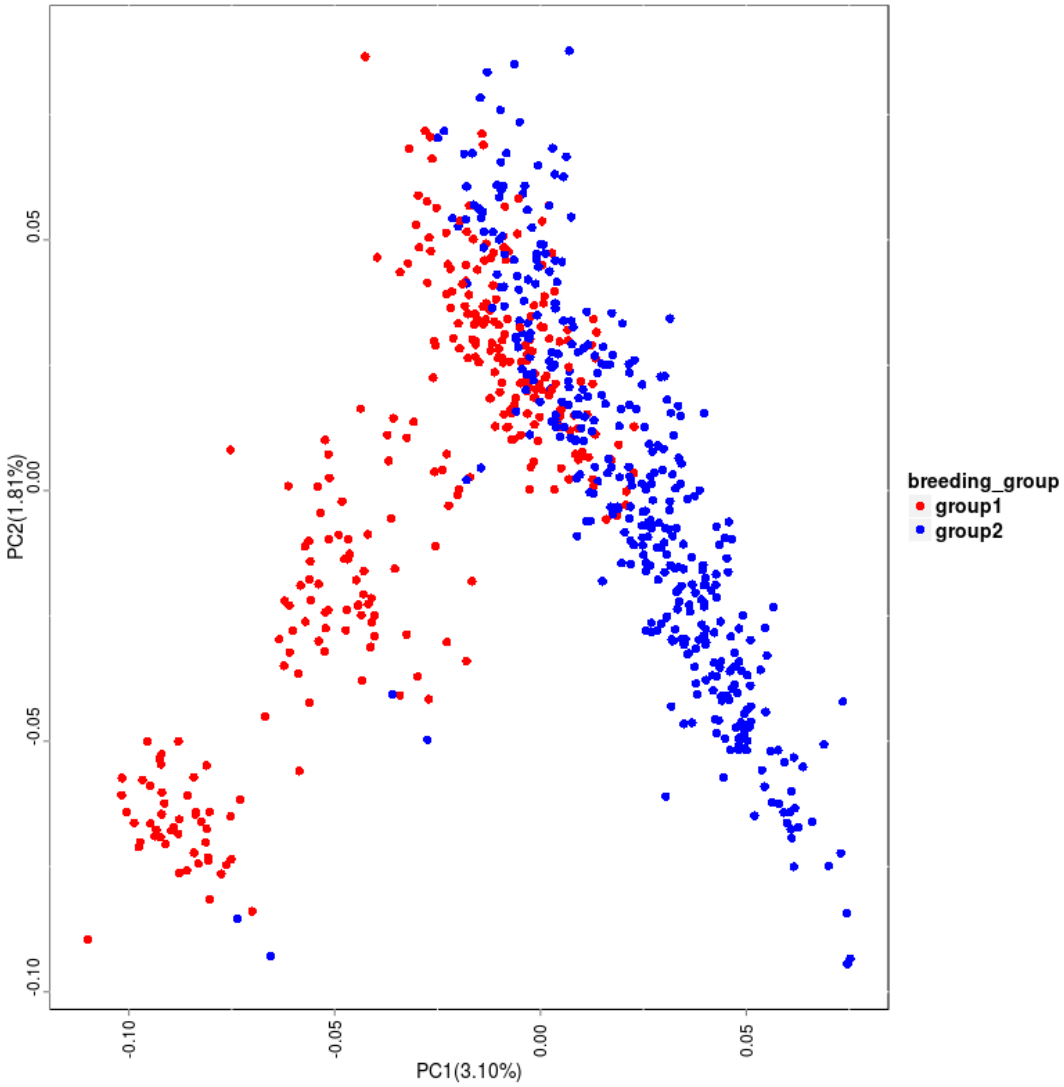

Principal component analysis of the genomic relationships among the genotyped bulls.

Breeding groups (blue and red dots) represent the grouping of genotyped animals based on a priori information related to two different artificial selection criteria applied in this *Bos indicus* (Nellore) population in the past 20 years.
